# Determining the clinical importance of treatment benefits for interventions for painful orthopedic conditions

**DOI:** 10.1186/s13018-014-0144-x

**Published:** 2015-02-03

**Authors:** Nathaniel P Katz, Florence C Paillard, Evan Ekman

**Affiliations:** Analgesic Solutions, 232 Pond Street, Natick, MA 01760 USA; Tufts University School of Medicine, 274 Tremont Street, Boston, MA 02111 USA; Appalachian Regional Orthopaedic & Sports Medicine Center, Appalachian State University Department of Athletics, 870 State Farm Road, Suite 100, Boone, NC 28607 USA

**Keywords:** MCID, MDC, Anchor-based methods, Distribution-based methods, Orthopedic surgery, Pain

## Abstract

The overarching goals of treatments for orthopedic conditions are generally to improve or restore function and alleviate pain. Results of clinical trials are generally used to determine whether a treatment is efficacious; however, a statistically significant improvement may not actually be clinically important, i.e., meaningful to the patient. To determine whether an intervention has produced clinically important benefits requires a two-step process: first, determining the magnitude of change considered clinically important for a particular measure in the relevant population and, second, applying this yardstick to a patient’s data to determine whether s/he has benefited from treatment. Several metrics have been devised to quantify clinically important differences, including the minimum clinically important difference (MCID) and clinically important difference (CID). Herein, we review the methods to generate the MCID and other metrics and their use and interpretation in clinical trials and practice. We particularly highlight the many pitfalls associated with the generation and utilization of these metrics that can impair their correct use. These pitfalls include the fact that different pain measures yield different MCIDs, that efficacy in clinical trials is impacted by various factors (population characteristics, trial design), that the MCID value is impacted by the method used to calculate it (anchor, distribution), by the type of anchor chosen and by the definition (threshold) of improvement. The MCID is also dependent on the population characteristics such as disease type and severity, sex, age, etc. For appropriate use, the MCID should be applied to changes in individual subjects, not to group changes. The MCID and CID are useful tools to define general guidelines to determine whether a treatment produces clinically meaningful effects. However, the many pitfalls associated with these metrics require a detailed understanding of the methods to calculate them and their context of use. Orthopedic surgeons that will use these metrics need to carefully understand them and be aware of their pitfalls.

## Introduction

Musculoskeletal conditions are often associated with reduced function and pain. In most common orthopedic conditions, patients and clinicians determine the success of treatment based on two main outcomes: pain reduction and functional improvement [1]. Whether a treatment is efficacious is determined by reviewing the results of randomized controlled trials (RCTs). The primary thrust of RCTs is to demonstrate a statistically significant difference in outcome between treatment groups. Statistical significance, however, does not necessarily imply *clinical significance*, or clinical importance, i.e., whether the observed improvement is meaningful to the patient [2-5]. Very small treatment effects, too small to be meaningful to patients, will be statistically significant if the sample sizes are large enough. Treatments that produce large and clinically important benefits may fail to achieve statistical significance if the sample size was too small. To this end, clinical researchers have introduced a series of metrics to determine whether improvements after treatment are clinically important [5].

Determining whether a treatment effect is clinically significant is paramount for a variety of stakeholders in the orthopedic community, including committees that develop treatment guidelines and formularies, make regulatory decisions, set market access and reimbursement policies, conduct systematic reviews, and practicing orthopedists who need to make informed decisions regarding patient treatments. Errors in determination of clinical significance of interventions for orthopedic conditions may have high consequences. These stakeholders bear a responsibility to understand how clinical importance is determined and ensure that decisions based on estimates of clinical importance are based on appropriate methods.

Other aspects of treatment should be considered in the choice of one orthopedic intervention over another. These include impact on physical function, quality of life (both psychological and social aspects), tolerability, convenience, availability, physician’s preference and experience, cost, and alternative treatment options [6]. This review focuses on the determination of the clinical importance of changes in pain intensity (PI), usually the primary outcome measure in clinical trials of these conditions.

Ultimately, this review should help those who generate the metrics of clinically important changes, and those who use these metrics to make informed decisions regarding orthopedic treatments. To this end, we will review how these metrics are generated, and how these metrics can and should be interpreted to make informed decisions, highlighting the many pitfalls of these two processes.

### Overview of the process

#### Two processes

**Figure 1:**
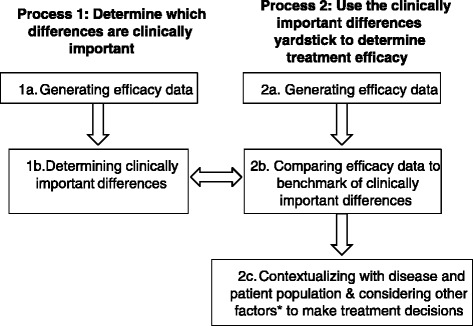
**Flow chart illustrating the overall process of determining CID and using it to determine treatment efficacy.** (Asterisk) Other factors include: disease, impact on quality of life, tolerability, convenience, availability, cost, and alternative treatments.

In determining whether an intervention studied in an RCT has produced a clinically important benefit, two different processes are involved (Figure 1). The first is to determine what magnitude of change in PI is considered clinically important in the relevant treatment population. To accomplish this goal, one needs to collect data from clinical trial evaluating treatments for pain in the specific condition (1a) and these data need to be analyzed to determine which changes in pain are considered clinically important. For example, one can determine through this process that a decrease in pain of 20 mm on the 0–100 mm visual analog scale (VAS) in a patient with knee osteoarthritis (OA) is a clinically important change.

The second process consists of using the metrics determined in the first process as a yardstick to determine whether a treatment has produced a clinically important change in a particular patient. For instance, an OA patient who has a decrease in pain of 30 mm on VAS after treatment is considered to have a clinically important response (as determined in the first process), i.e., the patient is considered as a responder to the treatment. Whether the treatment produces a clinically meaningful impact is then determined by comparing the proportion of “responders” on active treatment compared to control.

### Metrics of clinically important changes

The most widely used metrics of clinical important changes are described and defined in Table 1. They are typically generated by determining longitudinal improvement, i.e., comparing treatment outcome from pre- to post-treatment.

**Table 1:** **Metrics of clinically important changes**

**Metric**	**Definition**	**Notes**
Smallest detectable difference (SDD)	The amount of difference in an outcome measure for which anything smaller cannot be reliably distinguished from random error in the measurement	
Minimal detectable change (MDC)		
Minimal clinically important difference (MCID)	The smallest change or difference in an outcome measure between pre- and post-treatment perceived as beneficial (or detrimental) by the patient [7-9]	Can be used to measure improvement and worsening
Minimal clinically important change (MCIC)		
Minimal clinically important improvement (MCII)		Only measures improvements
Clinically important difference (CID)	The difference in outcome measure that is considered clinically important/meaningful	

**Figure 2:**
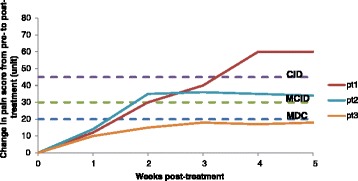
**Metrics of clinical importance**—**theoretical example for a pain measure.** MDC (blue dotted line), MCID (green dotted line), and CID (purple dotted line) are illustrated. The improvement of pain score over time (weeks) is shown for three theoretical patients (Pt1, Pt2, and Pt3). After week 4, the treatment is considered successful for Pt1 (change in pain > CID), the treatment is marginally successful for Pt2 (change in pain > MCID but < CID), and treatment failed in Pt3 (change in pain < MDC).

How do these metrics fit together? If the MDC on a pain scale was determined to be 20 units, the minimum clinically important difference (MCID) threshold 30 units, and the clinically important difference (CID) 45 units, this means that the pain scale cannot detect changes below 20 units, the *minimum* change in pain score considered clinically important by patients is 30 units, and the change in pain score considered clinically important is 45 units (Figure 2).

### Calculating the SDD, MCD, MCID, and CID

#### Data generation

To determine MCID in painful disorders, one needs to first collect clinical trial data with a pain endpoint. The clinical data used to make determinations of the smallest detectable difference (SDD)/MCID/CID should be as similar as possible (same population, same pain endpoint, similar treatment if applicable, similar study design) as the trial in which the MCID is used as a yardstick. This is because many factors can influence the effect size—and therefore the MCID—in a clinical trial.

First, as expected, the MCID is *specific* to the pain measure and scale used: an MCID calculated using 0–100 mm VAS pain is not equal or convertible to an MCID using the 0–10 numerical rating scale (NRS). Therefore, to determine whether the decrease in PI in a trial has reached the MCID, one needs to compare the trial results with MCID values calculated with the *same* pain scale. Tables 2 and 3 provide the MCID values calculated for different pain scales in different publications.

**Table 2:** **Example of calculations of MCID and CID in pain score in OA patients**

**Reference (treatment)**	**Method**	**Categories in anchor**	**Definition of MCID or CID for improvement**	**MCID or CID**
**Pain on VAS (0–100 mm scale)**				
Tubach 2005 (NSAIDs) [10]	Anchor—PGA	None, poor, fair, good, excellent	75th percentile of the change in score among patients whose evaluation of response to treatment (on PGA) was “good” (MCID)	−19.9 mm (knee) − 15.3 mm (hip)
**Pain on NRS (0–10 pt)**				
Farrar 2001 [11]	Anchor―PGIC	Very much improved, much improved, minimally improved, no change, minimally worse, much worse, very much worse	Decrease in pain score for patients “much improved” (CID)	2 pt
			Decrease in pain score for patients “very much improved” (CID)	≥4 pt
	ROC—PGIC	Very much improved, much improved, minimally improved, no change, minimally worse, much worse, very much worse	Decrease in pain score for patients “much improved” (CID)	≥1.7 pt
**Pain on WOMAC (on 0–100 pt scale, unless otherwise noted)**				
Ehrich 2000 (NSAIDs) [12]	Anchor—PGA	None, poor, fair, good, excellent	Difference btw “none” and “poor” response on PGA (MCID)	9.7 mm
Angst 2001 (inpatient rehabilitation) [13]	Anchor—transition question	Much worse, slightly worse, equal, slightly better, much better	Difference btw “equal” and “slightly better” groups (MCID)	0.75 pt^a^
Escobar 2007 (TKR) [14]	Anchor—transition question	A great deal better, somewhat better, equal, somewhat worse, a great deal worse	Difference btw baseline score and scores for patients declaring changes “somewhat better” (MCID)	23 pt
Quintana 2012 (THR) [15]^a^	Anchor—transition question	Seven items from “a great deal better” to “a great deal worse”	Mean change score for patients whose response was “a little better” (MCID)	15, 23, 36 pt^b^
	Anchor—PASS question	Totally satisfied, slightly satisfied, not satisfied, not at all satisfied	Mean change in score for the 75th percentile of patients in the probability curve reporting being totally satisfied or slightly satisfied (MCID)	20, 25, 25 pt^b^
	ROC—PASS question	Totally satisfied, slightly satisfied, not satisfied, not at all satisfied	Patients reporting being totally satisfied or slightly satisfied—optimal point on curve (MCID)	19, 25, 25 pt^b^
Escobar 2013 (TKR) [16]	Anchor—transition question	A great deal better, somewhat better, equal, somewhat worse, a great deal worse	Mean change in patients “somewhat better” (MCID)	30 pt
	ROC—transition question	A great deal better, somewhat better, equal, somewhat worse, a great deal worse	Mean change in patients “somewhat better” (MCID)	20–24 pt^c^
	Anchor—question about satisfaction	Very satisfied, somewhat satisfied, somewhat dissatisfied and very dissatisfied	Patient “somewhat satisfied” (MCID)	27 pt
**SF-36 bodily pain (0–100 pt scale)**				
Angst 2001 (inpatient rehabilitation) [13]	Anchor—“transition” questionnaire	Much worse, slightly worse, equal, slightly better, much better	Improvement MCID = Difference btw “equal” and “slightly better” (MCID)	7.8 pt for improvement
Escobar 2007 (TKR) [14]	Anchor—“transition” question at 6 months or 2 years	A great deal better, somewhat better, equal, somewhat worse, a great deal worse	Difference btw baseline score and scores for patients declaring changes “somewhat better” (MCID)	17 pt

*Btw* between, *PASS* Patient Acceptable Symptom, *THR* total hip replacement, *pt* point, *TKR* total knee replacement.

^a^On a 0–10 point scale.

^b^MCID is reported for different patients’ baseline pain, divided in tertiles.

^c^Data for one cohort (derivation cohort) only are shown.

**Table 3:** **Examples of calculation of MCID or CID in pain scores in patients with painful spine conditions**

**Reference (treatment)**	**Method**	**Categories in anchor**	**Definition of MCID or CID for improvement**	**MCID or CID** ^**a**^
**Distribution-based approaches**				
Carreon 2013 (lumbar fusion surgery) [17]	MDC	N/A—no anchor used	MDC defines the MCID	1.16 pt—back pain, 1.36 pt—leg pain
Gum 2013 (lumbar fusion surgery) [18]	MDC	N/A—no anchor used	MDC defines the MCID	0.20 pt—back pain, 0.23 pt—leg pain
**Anchor-based approaches**				
Gum 2013 (lumbar fusion surgery) [18]	ROC—health transition item on SF-36	Much worse, somewhat worse, about the same, somewhat better, and much better	Change in pain score for patients being “somewhat better” (MCID)	3.08 pt—back pain, 2.83 pt—leg pain
			Change in pain score for patients being “much better” (CID)	5.32 pt—back pain, 4.98 pt—leg pain
Carreon 2010 (cervical spine fusion) [19]	ROC—Health transition item of SF-36	Much better, somewhat better, about the same, somewhat worse, much worse	Distinguish the “somewhat better” from the “about the same” patients (MCID)	2.5 pt—arm and neck pain
Copay 2008 (lumbar spine surgery) [20]	Anchor—satisfaction with results scale	Answers: definitively true, mostly true, don’t know, mostly false, or definitively false to the following five items: 1. “I can do the things I thought I would be able to do after surgery”; 2. “I was helped as much as I thought I would be by my surgery”; 3. “My pain was reduced as much as I expected it to be after surgery”; 4. “The benefits of my care outweighed the setbacks it caused me”; 5. “All things considered, I would have the surgery again for the same condition”	Patients classified as “satisfied” and “don’t know”	1.2 pt—back pain, 1.6 pt—leg pain
Solberg 2013 (lumbar discectomy) [21]	Anchor—global perceived scale of change	Completely recovered, much improved, slightly improved, no change, slightly worse, much worse, and worse than ever	Patient reporting to be “completely recovered” or “much better” (CID)	2.5 pt—back pain, 3.5 pt—leg pain
Parker 2013 (anterior cervical discectomy and fusion) [22]	ROC—NASS	1) The treatment met my expectations; 2) I did not improve as much as I had hoped, but I would undergo the same treatment for the same outcome; 3) I did not improve as much as I had hoped, and I would not undergo the same treatment for the same outcome; and 4) I am the same or worse than before treatment	Patients with choice 1 classified as responders; choices 2–4 are non-responders (CID)	4.0 pt—VAS neck pain, 4.0 pt—VAS arm pain
	Combination: NASS anchor + MDC^b^			2.6 pt—VAS neck pain, 4.1 pt—VAS arm pain

*NASS* North American Spine Society patient satisfaction scale, *N/A* not applicable.

^a^All MCID and CID data presented in this table are for the 0–10 NRS, except for Parker 2013 which uses a 0–10 mm VAS.

^b^The MDC approach defines the MCID value as the upper value of the 95% CI for the average change score seen in non-responders (defined based on the anchor).

Interestingly, the MCID does not depend on the type of treatment [11,12,23]. For instance, the MCID on VAS for pain was ~25 mm in OA patients regardless of treatment (NSAIDs, THR, TKR, or rehabilitation) [11], i.e., improvement is improvement regardless what produced it.

MCID may not be the same across diseases unless this has been demonstrated. Stauffer et al. [23] found that the MCID in VAS pain is different between patients with knee OA, hip OA, and back pain, while Farrar et al. [11] found similar MCID for pain NRS in patients in OA, painful diabetic, neuropathy, postherpetic neuralgia, low back pain, and fibromyalgia.

The MCID also varies with the severity of the disease: the MCID is larger at higher baseline pain levels, suggesting that patients with severe pain require a larger amount of pain relief to report being satisfied (Table 4) [23]. This is corroborated by data from Quintana [15] and Farrar [11] (Table 2).

**Table 4:** **MCID by patient population and tertile of baseline VAS score**

**Patient population**	**Baseline pain on VAS 0–100 mm**		
	**30–49 mm**	**50–65 mm**	**>65 mm**
Knee OA	11	27	37
Hip OA	7	24	30
Low back pain	9	19	29

Data are MCID for improvement in pain (VAS 0–100 mm).

Data are from Tubach et al. [10].

### Calculating SDD and MDC

There are two methods of estimating SDD/MDC which are purely statistical [23]: SDD is calculated using the limits of agreement method (a.k.a., the Bland and Altman method [24]) and MDC using the standard error of the mean (SEM). One problem with the SDD is that its value depends on the sensitivity of the statistical method used [25].

### Calculating MCID or CID

There are four methods to calculate the MCID: consensus, anchor-based, distribution-based, or a combination of anchor- and distribution-based methods.

#### Consensus methods

In the consensus method, a panel of expert is convened and each expert provides his/her best estimate of the MCID until a consensus is achieved. For instance, the consensus method was used to define a decrease in PI from a baseline of ≥20% (moderate improvement) as a MCID and a decrease of ≥30% or ≥50% as a CID [26]. A ≥50% reduction in pain from the baseline corresponds to “very much improved” on the Patient Global Impression of Change (PGIC), providing a cross-validation of the consensus method [11]. The main problem with the consensus method is that it is not based on the opinion of the patient.

#### Anchor-based methods

In the anchor-based method, the pain scores are compared with an external, independent, face valid criterion (termed “anchor”) to determine a MCID in pain. The anchor is generally a questionnaire in which patients report their level of improvement, chosen based on its good reliability and validity. Examples of anchors include the PGIC, a “transition” question, the Patient Global Assessment (PGA) of treatment effectiveness (Tables 2 and 3). Using these anchors, MCID can be calculated for both improvement and worsening (most studies report the MCID for improvement in pain).

**Figure 3:**
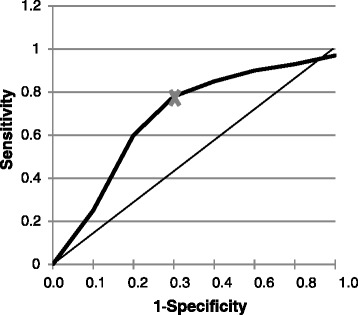
**Calculating CID by the ROC method.** This analysis defines the sensitivity and specificity of different cutoffs of a predictor (e.g., pain) for an anchor (e.g., global change). The diagonal line represents a test with no predictive value. The curve is the ROC analysis. The CID is the cutoff of the predictor with the highest sensitivity and specificity for predicting the anchor, i.e., the upper left most point on the ROC curve (marked by an *x* on the graph). For example, the *x* might represent 30% pain reduction as the best cutoff to predict “much improved” on a PGIC.

The anchor can be used to create a receiver operated characteristics (ROC) curve, a common approach for determining the performance of diagnostic tests. The ROC cutoff is the value for which the sum of % of false positives and false negatives is the smallest (as illustrated in Figure 3). Alternatively, the cutoff is the value for PI reduction that best predicts a specified change in the anchor (e.g., patients “much improved” on a PGIC). Tables 2 and 3 show examples of anchor-based calculations of MCID or CID in pain in OA (Table 2) and in painful spine conditions (Table 3).

Calculation of the MCID versus CID is based on the anchor threshold. Calculating the MCID is based on selecting patients with *minimum* improvement on the anchor, while calculating the CID is based on selecting patients with *great* improvement. For instance, Gum [18] defined the MCID for 0–10 NRS as the mean change in PI in patients reporting feeling “somewhat better” and the CID as the change in PI in patients “much better” (Table 3); with these cutoffs, patients with a decrease in back PI of ≥3.1 points on the 0–10 NRS can be considered as minimally improved (MCID), while those with a decrease in PI of ≥5.3 points can be considered greatly improved (CID) (Table 3).

There are several problems associated with anchor-based approaches. First, they are not suitable for conditions where most patients will improve and only a few remain unchanged. Second, they do not take into account the variability of the sample. The optimal cutoff is an average; some patients require smaller or larger changes in PI, for example, to feel “much improved.” Third, there are inherent problems associated with anchors for pain: (i) there is no *objective* measure of improvement in pain since pain is a subjective experience; (ii) the anchors used often measure “improvement,” which is a composite endpoint of pain and other factors, such as function; and (iii) patients may have difficulty to recall their initial status to accurately report their level of improvement between pre- and post-treatment. Finally, and most importantly, the degree of change in the anchor that is meaningful to patients is usually uncertain, defeating the purpose of the anchor.

#### Distribution-based methods

Distribution-based methods use a statistical method to measure the variability of a variable (e.g., PI) within the sample and determine what degree of change in that variable is generally of clinical importance. One distribution-based approach uses the SEM: because the SEM value reflects the imprecision of the measurement, an MCID value below the SEM does not reflect a true change. Another distribution-based method is the MDC, where MDC is the smallest change above the measurement error. In this approach, the MCID value is defined as the upper value of the 95% confidence interval (CI) for the average change score in non-responders. The basis of using the SEM as MCID is supported by the fact that MCIDs often turn out to be around 1 SEM or ½ SD [27].

A few studies in painful orthopedic conditions have used distribution-based methods to calculate the MCID ([17,18]; Table 3).

Distribution-based approaches have two important limitations. First, they allow calculation of the MCID, but not the CID. Second, they only define the minimum value below which a change in pain score is hopefully not due to measurement error.

#### Combination of anchor-based and distribution-based methods

One approach that combines both methods consists of (i) defining responders/non-responders using an anchor for improvement and then (ii) calculating the MCID based on the MDC: the MCID is then equal to the upper value of the 95% CI for the average change in pain score seen in non-responders with the anchor. Using this combined method, Parker et al. found a MCID of 2.6 points for neck pain on VAS, compared to 4.1 points for the ROC method [22]; the MCID for arm pain, however, was similar with both methods (4.1 vs. 4.0 points, respectively). Another way to do this is to use graphical method that integrates both anchor- and distribution-based approaches (see de Vet [28] for details). Using that approach, de Vet [28] found an MCID of 4 points on 0–10 NRS pain with the combined approach, versus 2.5 points with the ROC approach alone. Although the combined approach is more complex, it has the advantage over the pure anchor-based method of accounting for the variability of the sample.

#### Pitfalls with the calculation of the CID

The CID value is dependent on the methodology used to calculate it. One study used a variety of anchor-based (average change, change difference, and ROC) and combination (anchor + SEM or MDC) methods and found that the MCID could vary by seven- to eightfold for the same anchor depending on the method used (Table 4) [20]. Another study that used four anchor-based or combination methods to calculate the MCID in pain in lumbar surgery found a wide range of MCID values, from 2.7 to 4.0 points for neck pain and from 2.4 to 4.2 points for arm pain on a 0–10 VAS [22]. Finally, a study found that the distribution-based MDC method yielded MCID values ten times lower than an anchor-based method (0.20 and 0.23 points by MDC vs. 3.1 and 2.8 points by anchor for back and leg pain, respectively) (Table 3) [18]. These studies clearly demonstrate that the MCID value is dependent on the method used to calculate it. However, this is not always the case, and some studies have shown that two different methods can yield similar MCID values ([11] [Table 2]; [15] [Table 2]; [22] [Table 3] for arm pain only).

The MCID calculation using the anchor-based method may also depend on the anchor type (PGIC, PGA, transition question, etc.). Various studies have compared the MCID values with two anchors (Tables 2 and 4). While in some instances, the anchor impacts the MCID ([15] [Table 2]), it has little effects in other cases ([14] [Table 2]; [20] [Table 5]). Thus, the MCID cannot be assumed to be robust to different methods of calculation.

**Table 5:** **MCID values determined by different methods**

	**Back pain**	
**Method of MCID determination**	**Anchor: HTI** ^**a**^	**Anchor: satisfaction** ^**b**^
Anchor-based		
Average change	2.9	3.4
Change difference	1.4	1.9
ROC	2.5	2.5
Combination of anchor-based and distribution-based methods		
SEM	0.4	0.4
MDC	1.2	1.2

From Copay 2008 [20].

^a^The HTI questionnaire asks patients to compare their health after treatment versus before treatment; the HTI answers are much better, somewhat better, about the same, somewhat worse, and much worse. Patients reporting being “somewhat better” or “about the same” were selected.

^b^The satisfaction questionnaire has five items (statements): “I can do the things I thought I would be able to do after surgery”; “I was helped as much as I thought I would be by my surgery”; “My pain was reduced as much as I expected it to be after surgery”; “The benefits of my care outweighed the setbacks it caused me”; “All things considered, I would have the surgery again for the same condition” to which patients can answer: definitively true, mostly true, don’t know, mostly false, or definitively false. Patients classified as “satisfied” and “don’t know” were selected.

Finally, the CID value depends on the threshold of improvement. For instance, selecting patients “much improved” versus “very much improved” on PGIC (anchor) doubled the CID value from 2 to ≥4 points [11].

### Using the MCID to determine treatment efficacy

#### Contextualizing the use of MCID as a yardstick

To appropriately use the MCID to determine the efficacy of a treatment in a trial, one must use MCID values that were calculated in a *very similar context* to the trial, i.e., for the same pain measure, in a similar patient population (pain condition, severity of disease at baseline, baseline characteristics), and using a similar trial design because the MCID is dependent on all these factors, as discussed above. In addition, one should be mindful of how the MCID was calculated, as many factors influence the MCID value, as discussed above (“Pitfalls in calculation and interpretation of clinically important differences” section). MCIDs values for a variety of pain measures, painful conditions, and patient populations have been calculated and published. To find the appropriate MCID yardstick to use among these publications, clinicians should search through publication databases using search words such as “MCID,” “pain,” accompanied with the treatment of interest (e.g., lumbar surgery), and the measurement of interest (e.g., VAS neck, back, or leg pain). Then, it requires sorting through these publications to find the most appropriate one (“Pitfalls in calculation and interpretation of clinically important differences” section). For instance, if one is interested in finding the MCID value for VAS neck pain in a population of patients undergoing anterior cervical discectomy and fusion, a type of search as described above retrieved a very appropriate article from Parker et al. [22], which shows an MCID value of 2.6 points.

Pitfalls in calculation and interpretation of clinically important differences

- Pitfalls in generating efficacy data to calculate metrics of clinically important differences
  - Choice of pain measure: different pain measures yield different MCIDs
  - Efficacy in clinical trials is impacted by various factors (population characteristics, trial design)
- Pitfalls in calculating the metrics of clinically important differences
- The MCID value is impacted by:
  - The method used to calculate it (anchor, ROC, distribution)
  - The type of anchor chosen
  - The definition (threshold) of improvement
  - The population characteristics such as disease type and severity (baseline pain score)
- Pitfalls in applying the metrics of clinically important differences to clinical trial data
  - The appropriate MCID value has to be found or calculated for the specific application
  - In clinical trials, the MCID yardstick should be applied to changes in individual subjects, not to group changes; applying individual CIDs on a group level is misleading

If the appropriate MCID value cannot be found, one approach is to calculate the MCID *within the same* clinical trial. In other words, the same clinical trial data serve to calculate the MCID value, which is then applied to each patient of the trial to determine which patient met the MCID threshold. For instance, a clinical trial of lumbar discectomy was used (i) to calculate the MCID of leg and back pain (3.5 and 2.5 points for leg and back pain, respectively, on 0–10 NRS) and (ii) to determine the proportion of patients with improved leg and back pain (i.e., patients with pain scores above the MCID) [21]. Using the ROC method, the authors found a MCID of 2.5 for NRS back and 3.5 for NRS leg; when applied to the trial data, the proportion of patients who had clinically significantly improvement (met the MCID) in leg pain and back pain was 67% and 59%, respectively.

### Comparing the efficacy of two treatments in clinical trials

The MCID can be used to determine treatment efficacy or to compare the efficacy of two active treatments in clinical trials. To do so, first, one has to calculate the proportion of patients in each treatment group that meet the MCID, defined as *individual patients for whom the difference between pre- versus post-treatment pain score is equal to or greater to the MCID threshold*. Then, the treatment groups can be compared for the proportion of patients who meet the MCID using a standard statistical method. For instance, a study evaluating transsacral axial interbody fusion found that only 50% of patients met the MCID of the VAS score for back pain [29]. Another study evaluating lumbar spine surgery found that 67% of patients met the MCID of leg pain [21].

One important error that is made when determining treatment efficacy in clinical trials is to calculate the *mean* difference (pre- versus post-treatment) pain score for the treatment group and comparing it to the MCID (“Pitfalls in calculation and interpretation of clinically important differences” section). The MCID is a metric that is based on longitudinal differences in *individual* patients and should be used in the same context.

Although this seems straightforward, the clinical trial data used to calculate the MCID have a few issues that are inherent to all analgesic trials: (i) analgesics that are known to be effective only result in an *average* decrease in PI scores of 0.5 to 1 point on a 0–10 NRS compared to placebo, which means that MCID values tend to be small (10–20 mm on VAS, Table 2); (ii) the proportion of responders (patients whose PI decreases by ≥30%) is often 40% to 50% for effective analgesics, which means that the % of patients who meet the MCID should be relatively low; (iii) the placebo effect is often high, leading to a situation whereby known effective analgesics frequently demonstrate non-significant effects compared to placebo (failed trials); and (iv) pain is a subjective experience and pain reporting by patients may vary according to patient characteristics and study design or conduct, affecting the pain endpoint and therefore the % patients who meet the MCID.

### Determining treatment efficacy in individual patients

The SDD/MDC and MCID can be used by orthopedic practitioners in the clinical setting to determine whether an individual patient has responded to treatment based on the patient’s *change in pain score between pre- versus post-treatment*. Figure 2 provides examples of three different cases with different response to a treatment. Categorizing patients by level of response to treatment should help physicians make further treatment decisions for patients (continue or change treatment) and make treatment decisions for other patients with a similar condition.

There are important caveats to applying the MCID, which is determined in clinical trial, to the clinical setting (“Pitfalls in calculation and interpretation of clinically important differences” section). First, the MCID is derived in a homogeneous population in clinical trial, while patients in the clinical setting are heterogeneous. Second, categorizing patients as responder/non-responder can negatively affect patient management as it does not take into account the category of patients who are borderline responders or poor responders. For these suboptimal responders, making an appropriate treatment decision will require additional clinical information regarding treatment effect.

One key problem in using the MCID in the clinical setting is that patients may not have clinically important changes *in pain* but may have substantial improvements in function, sleep, or other clinical benefits. Thus, applying only MCID in painful orthopedic indications where function and other outcomes are important to make treatment decisions may not be the best approach, i.e., may have negative implications for patient management. Other domains including physical functioning, emotional functioning, and overall improvement, for which an MCID can be calculated, should be considered. Adverse effects, burden to patient, and cost should also be taken into consideration when making treatment choices (“Pitfalls in calculation and interpretation of clinically important differences” section).

### Using the MCID to inform guidelines

The MCID (or minimal clinically important improvement (MCII)) derived from clinical trials is used to inform guidelines. For example, in the OA treatment guidelines [30], clinical significance takes into account both the statistical significance of the treatment efficacy (versus placebo) and the 95% CI of the mean pain score relative to MCII (Table 6) to flag as ineffective treatments whose upper bound estimate of treatment efficacy remains below the MCII threshold.

**Table 6:** **Level of efficacy used in OA treatment guidelines depending on the statistical and clinical importance (MCII) of the treatment effect**

**Descriptive term**	**Condition of use**
Clinically significant	Statistically significant and lower limit of CI > MCII
Possibly clinically significant	Statistically significant and CI contains the MCII
Not clinically significant	Statistically significant and upper limit of CI < MCII
True negative finding	Not statistically significant and upper limit of CI < MCII
Inconclusive finding	Not statistically significant but CO contains the MCII

From AAOS 2013 [30].

The use of the MCID or MCII as the cornerstone to determining treatment efficacy in guidelines has many caveats [31]. First, the MCID calculation can greatly vary between studies, making the comparison between treatments assessed in different studies very difficult. Second, many clinical variables (disease severity, age, body weight, activity level, comorbidities, and prior treatments) not reflected in the MCID are important to consider when selecting a treatment for a patient. Thus, the MCID should only be considered as a tool to analyze clinical trial results that supplements, but does not replace, expert clinical judgment to make decisions when treating patients with orthopedic conditions.

## Conclusions

The MCID and CID are useful tools to determine whether a treatment produces clinically meaningful effects in a patient. However, these metrics have many pitfalls, requiring a good understanding of the methods to calculate them and their context of use. Orthopedic surgeons that will use these metrics need to carefully understand them and be aware of their pitfalls.
